# Microcapsule-Type Self-Healing Protective Coating That Can Maintain Its Healed State upon Crack Expansion

**DOI:** 10.3390/ma14206198

**Published:** 2021-10-19

**Authors:** Ji-Sun Lee, Hyun-Woo Kim, Jun-Seo Lee, Hyun-Soo An, Chan-Moon Chung

**Affiliations:** Department of Chemistry, Yonsei University, Wonju 26493, Korea; leej_s@yonsei.ac.kr (J.-S.L.); skyna123@yonsei.ac.kr (H.-W.K.); leejs19@yonsei.ac.kr (J.-S.L.); hyunsoo.an@yonsei.ac.kr (H.-S.A.)

**Keywords:** microcapsule, self-healing coating, crack expansion, maintaining the healed state

## Abstract

The purpose of this study was to develop a microcapsule-type self-healing coating system that could self-heal cracks and then maintain the healed state even upon crack expansion. Mixtures consisting of a photoinitiator and two methacrylate components, bismethacryloxypropyl-terminated polydimethylsiloxane (BMT-PDMS) and monomethacryloxypropyl-terminated PDMS (MMT-PDMS), were transformed into viscoelastic semi-solids through photoreaction. The viscoelasticity of the reacted mixtures could be controlled by varying the mass ratio of the two methacrylates. Through a stretchability test, the optimal composition mixture was chosen as a healing agent. Microcapsules loaded with the healing agent were prepared and dispersed in a commercial undercoating to obtain a self-healing coating formulation. The formulation was applied onto mortar specimens, and then cracks were generated in the coating by using a universal testing machine (UTM). Cracks with around a 150-μm mean width were generated and were allowed to self-heal under UV light. Then, the cracks were expanded up to 650 μm in width. By conducting a water sorptivity test at each expanded crack width, the self-healing efficiency and capability of maintaining the healed state were evaluated. The B-M-1.5-1-based coating showed a healing efficiency of 90% at a 150-μm crack width and maintained its healing efficiency (about 80%) up to a 350-μm crack width. This self-healing coating system is promising for the protection of structural materials that can undergo crack formation and expansion.

## 1. Introduction

A coating is usually applied to the surface of structural materials, such as concrete and steel, to protect it from corrosive substances including water, carbon dioxide, and chloride ions. However, damage to the protective coating can occur by microcracking or scratching, which causes the penetration of the corrosive substances through the damaged region. If a self-healing ability to repair the damage by itself is introduced into the protective coating, it can effectively protect the materials from deterioration. Self-healing coating technology can contribute to the extension of the material’s lifetime, the reduction of maintenance expenses, and the enhancement of public safety [1,2,3].

Microcapsule-type self-healing protective coatings have been developed extensively by incorporating microcapsules loaded with a variety of healing agents [4]. When the self-healing coating is damaged, the microcapsules in the damaged region rupture, and the healing agent is released. The healing agent fills the damaged region and undergoes a chemical reaction triggered by UV light [5,6,7], a catalyst [8,9], atmospheric oxygen [10,11], moisture [12], or a crosslinking agent [13]. In most cases of previous studies on microcapsule-type self-healing coatings, the released healing agent chemically transformed into a hard solid. 

Essentially, the conventional microcapsule-type self-healing system is limited to a single local self-healing event; if damage occurs in the healed region again, then self-healing cannot be repeated, not only because the microcapsules have been depleted, but also because the solidified healing agent has become a hard solid with no additional self-healing capability [1,14]. To solve this problem, we developed a more advanced microcapsule-type self-healing system that is highly resistant to secondary damage in the healed region [13,14]. In these systems, the released healing agent is transformed into a viscoelastic substance that is not damaged by stress, such as intense vibrations (self-healing coatings on bridges and tunnels may be exposed to vigorous vibrations).

On the other hand, another aspect that was considered is that initial cracks tend to gradually expand (i.e., the crack width increases) [15,16]. Even if the cracks were successfully self-healed first, secondary cracks could occur in the healed region where the cracks expanded. The objective of the present study was to develop a microcapsule-type self-healing coating system that could self-heal the initial cracks and then maintain the healed state, even upon crack expansion. Our strategy was to develop a healing agent that transformed into a viscoelastic substance that could heal the primary cracks and then be sufficiently stretched without being torn by crack expansion. A mixture of bismethacryloxypropyl- and monomethacryloxypropyl-terminated polydimethylsiloxane (BMT-PDMS and MMT-PDMS) was used as a healing agent (Scheme S1), and the viscoelasticity of its reaction product was controlled by varying the mass ratio. Methacryloxypropyl-terminated PDMSs have been used in different applications as self-healing coatings [5,6], hydrogels [17,18], imprinting materials [19,20], and polymerization stabilizers [21], based on their hydrophobicity and reactive methacryloxy groups. Microcapsules loaded with the healing agent were prepared and were dispersed in the coating formulation to obtain a self-healing coating. The coating was applied onto mortar specimens, and cracks were generated in the coating. By conducting a water sorptivity test, the self-healing efficiency and capability of maintaining the healed state of the coating system were evaluated.

## 2. Materials and Methods

### 2.1. Materials

Urea, aqueous formaldehyde solution (37 wt%), and resorcinol were used as microcapsule shell materials and were purchased from Merck-Korea (Seoul, Korea). Poly (ethylene-*alt*-maleic anhydride) (EMA) and 1-octanol were used as a surfactant and ant-forming agent, respectively, and were purchased from Merck-Korea. Ammonium chloride for use as curing agent was purchased from Duksan Pure Chemical (Seoul, Korea). Bismethacryloxypropyl-terminated polydimethylsiloxane (BMT-PDMS) (molecular weight: 25,000 g/mol; viscosity: 1000 cSt) and monomethacryloxypropyl-terminated polydimethylsiloxane (MMT-PDMS) (molecular weight: 1000 g/mol; viscosity: 150–200 cSt) for use as healing agents were purchased from Gelest (Morrisville, PA, USA). The chemical structures of BMT-PDMS and MMT-PDMS are shown in Scheme S1. Benzoin isobutyl ether (BIE) for use as a radical photoinitiator was purchased from Tokyo Chemical Industry (Tokyo, Japan). A fluorescent fluid (OIL-GLO 44-P) for use as a fluorescence indicator was purchased from Spectroline (Melville, NY, USA). Water, cement, and sand were mixed with a mass ratio of 1:2:6 according to the KS F 2476 and KS F 7936 standard methods. The mixture was poured into a mold to prepare 40 mm × 40 mm × 120 mm square pillar mortar specimens. Acrylic undercoating and top-coating formulations (Wrapping Coat^®^) were donated by Samjoongcnc Co. (Pochun, Korea).

### 2.2. Instruments

Photoirradiation was conducted using an exposure system (NEXSAL, Hantech, Gunpo, Korea) equipped with a xenon short arc lamp (light intensity: 22.7 mW/cm^2^) in conjunction with a UV cutoff filter (>305 nm; Edmund Optics Co., Barrington, IL, USA). The IR spectra were recorded on a Fourier transform infrared (FT-IR) spectrophotometer (Spectrum One B, Perkin Elmer Co., Waltham, MA, USA). A mechanical stirrer (NZ-1000, EYELA, Tokyo, Japan) equipped with a propeller-type impeller was used in the microencapsulation. An advanced rheometric expansion system (ARES, Rheometric Scientific, Piscataway, NJ, USA) was used to measure the viscoelasticity of the products of the reaction mixtures. An optical and fluorescence microscope (BX51, Olympus, Tokyo, Japan) was used to observe the prepared microcapsules, stretched film, and coating surface. Microcapsule size was analyzed using a CCD camera (CC-12, Olympus, Tokyo, Japan) in the microscope and image analysis software (analysis TS, Olympus, Tokyo, Japan). A universal testing machine (UTM, QC-505M1, Cometech Testing Machines, Taichung, Taiwan) was used to generate and expand cracks in the mortar specimens using a three-point bending test method. A digital microscope (B008, Supereyes, SKL Technology, Kwun Tong, Hong Kong) was used to measure the maximum stretched width (MSW) and the crack width. The MSW and crack width were measured at 10 points along the length direction of each stretched film or crack, and the values were averaged.

### 2.3. Selection of the Healing Agent

In order to find the optimal composition of the healing agent, BMT-PDMS and MMT-PDMS were mixed at various mass ratios (Table 1). The two methacrylates, BIE (1 wt%), and the fluorescent fluid (1 wt%) were mixed by vortexing for 5 min. An amount of 0.01 mL of each mixture was applied on an end side of a glass specimen (10 mm × 50 mm × 5 mm), and this end side was placed in contact with an end side of another glass specimen (Figure S1). After the photoirradiation of the mixture for 20 min, each glass specimens was slowly pulled in the opposite direction until the stretched film of the mixture started to tear. The stretchability test was recorded using a portable digital microscope, and the MSW before the tear was measured.

### 2.4. Microencapsulation

In a 100 mL beaker, 2.5 wt% aqueous solution of EMA (5 mL) was added to water (20 mL) and urea (0.503 g). Then, resorcinol (0.050 g) and ammonium chloride (0.050 g) were added. The beaker was placed in a water bath maintained at 30 °C, and the solution was stirred at 300 rpm using a mechanical stirrer. The pH was adjusted to about 3.5 by using a 10 wt% NaOH aqueous solution. The stirring speed was increased to 1000 rpm, and 2–3 drops of 1-octanol were added to remove the surface bubbles generated during stirring. An amount of 6 mL of core material (a mixture of BMT-PDMS, MMT-PDMS, and BIE with 59.4:39.6:1 mass ratio) was added, and stirring was continued for 30 min to form a stable emulsion (the core material was heated in advance at 30 °C to reduce its viscosity). An amount of 37 wt% formaldehyde solution (1.456 g) was added into the beaker, and the temperature of the water bath was raised to 60 °C and was maintained for 4.5 h. During the microencapsulation, the pH gradually decreased, and the final pH value was measured to be about 2.6. The reaction mixture was cooled to room temperature and was filtered using vacuum filtration. The obtained microcapsules were washed with distilled water and THF, and they were then air-dried for 24 h.

### 2.5. Evaluation of Primary Self-Healing Function by Water Sorptivity Test

The microcapsules were dispersed in a commercial undercoating formulation at a concentration of 50 wt%. To coat an area of 1600 mm^2^ on a mortar specimen, undercoating and top-coating formulations of 0.3 g each were used. The self-healing undercoating containing microcapsules was applied onto mortar specimens (Figure S2). After drying, the top-coating was applied. Cracks were generated in the surface of the applied mortar by pressing the center of the opposite side of the coated side using UTM in the three-point bending test mode. Initially, cracks with a 150 ± 5 µm width were generated. The cracked regions of the mortars were photoirradiated with a xenon lamp for 2 h to allow for a sufficient crosslinking reaction. After weighing the mortar specimen, the mortar side with the coating material applied was immersed in water. After 10 h, surface water was removed, and the increase in mass due to water uptake was measured. At least three water uptake values were obtained and averaged. A similar test was conducted using a control coating without microcapsules. The healing efficiency was calculated using the following Equation (1):

Healing efficiency (%) = (1 − U_self-healing_/U_control_) × 100 (1)

where U_self-healing_ and U_control_ are the water uptake of the mortar specimens with the self-healing and control coatings, respectively. 

### 2.6. Evaluation of the Capability to Maintain the Healed State

The self-healing coating samples were prepared by coating mortars, crack generation, and photoirradiation, as described in Section 2.5. The primary cracks with a 150-µm width were expanded to a 200, 250, 300, 500, or 630-µm width by UTM. The width of the crack was adjusted by varying the speed and force in the three-point bending test mode. The water sorptivity test was conducted as described above, and the healing efficiency was calculated using Equation (1). A minimum of at least three water uptake values were obtained and averaged.

## 3. Results and Discussion

### 3.1. Preparation and Characterization of the Healing Agent

**Figure 1:**
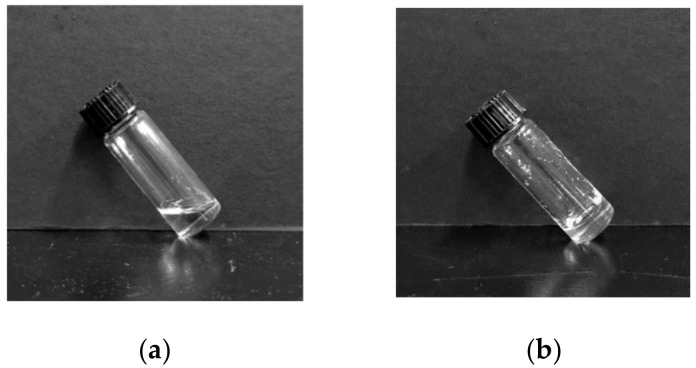
Photographs of B-M-1-1 in a 5-mL vial: (**a**) before and (**b**) after UV irradiation.

In order to find the optimal composition of the healing agent, BMT-PDMS and MMT-PDMS were mixed at various proportions and were polymerized (Scheme S1). Each mixture contained 1 wt% BIE as a radical photo-initiator. As shown in Figure 1, before photoirradiation, the mixtures were viscous liquids, and after photoirradiation, some of the samples were transformed into semi-solids (Table 1). The other samples that had higher contents of MMT-PDMS maintained a liquid state. The formation of the semi-solids was attributed to the higher crosslinking density that originated from the higher content of the bismethacrylate, BMT-PDMS, in comparison to the other liquid products.

**Figure 2:**
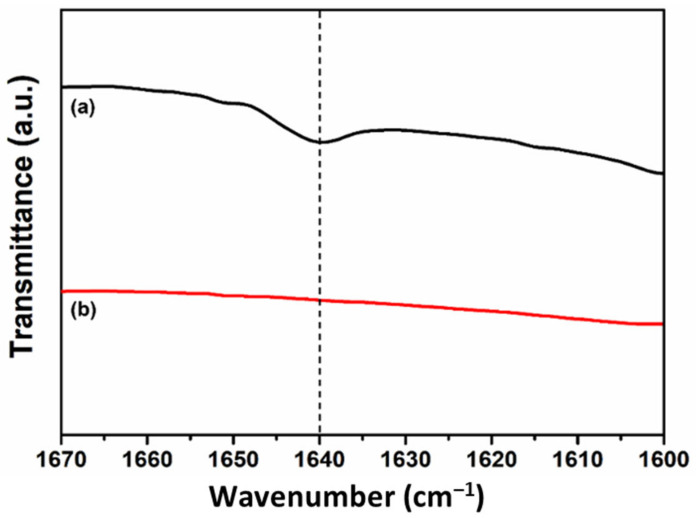
FT-IR spectra in the C=C region for B-M-1-1: (**a**) before and (**b**) after photoirradiation.

The photoreaction behavior of the mixtures was investigated using FT-IR spectroscopy (Figure 2 and Figure S3). The samples showed a methacrylate C=C stretching vibration band at 1640 cm^−1^ (Figure 2a). The absorption band disappeared after photoirradiation, indicating that the reaction of the methacrylate C=C groups was complete. The photocrosslinking reaction would have occurred because the mixtures contained the bismethacryl BMT-PDMS.

**Figure 3:**
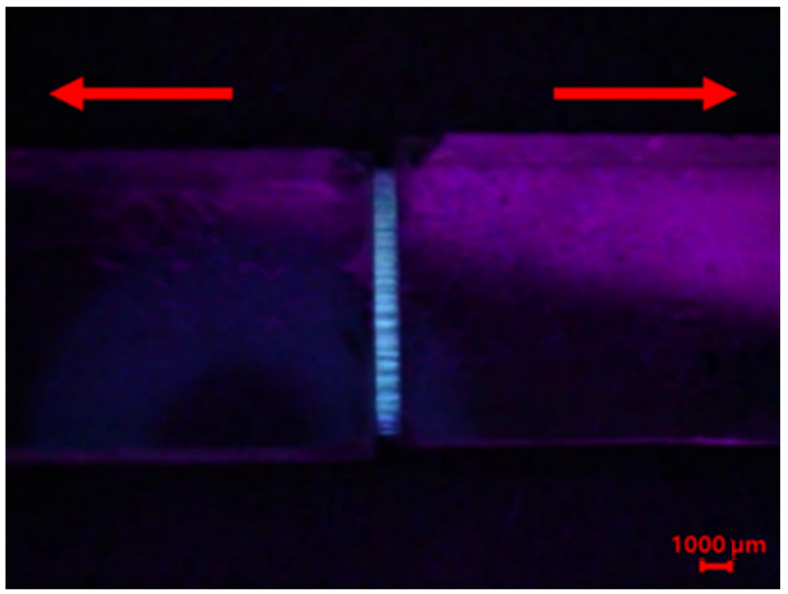
A fluorescence photograph of stretched B-M-1.5-1 after the photoreaction. The B-M-1.5-1 sample contained 1 wt% of a fluorescent indicator, and the photograph was taken under 450–480-nm light.

**Figure 4:**
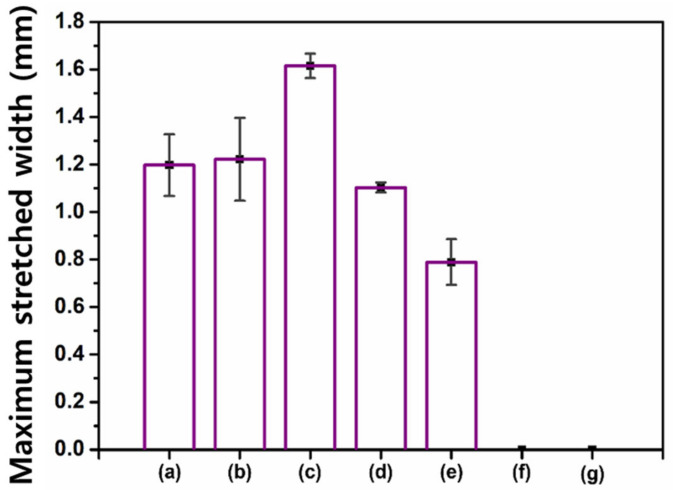
Maximum stretched widths (MSWs) of the photoreacted mixture of BMT-PDMS and MMT-PDMS: (**a**) B-M-3-1; (**b**) B-M-2-1; (**c**) B-M-1.5-1; (**d**) B-M-1-1; (**e**) B-M-1-1.5; (**f**) B-M-1-2; and (**g**) B-M-1-3.

The optimal composition of the healing agent was determined using a stretchability test. Each mixture sample was placed between the end sides of two glass slides and photoirradiated, as shown in Figure S1. By slowly pulling of each glass slide, the reaction product was stretched to form a film (Figure 3). For facile observation, 1 wt% of a fluorescent indicator (OIL-GLO 44-P) was added to the original reaction mixture. The pulling was stopped when the reaction product started to tear. The maximum stretched width (MSW) without the products tearing was measured. The MSWs according to the mass ratio of BMT-PDMS and MMT-PDMS are shown in Figure 4 and Table S1. The MSW increased and then decreased as the proportion of MMT-PDMS increased. In the cases of the reaction products of B-M-1-2 and B-M-1-3 having high contents of MMT-PDMS, the MSW could not be measured because these products were highly flowable. The MSW of the reacted B-M-1.5-1 was determined to be 1.62 mm, which was the widest among the samples. On the basis of these results, it was confirmed that B-M-1.5-1 was the most suitable healing agent for the purposes of this study.

**Figure 5:**
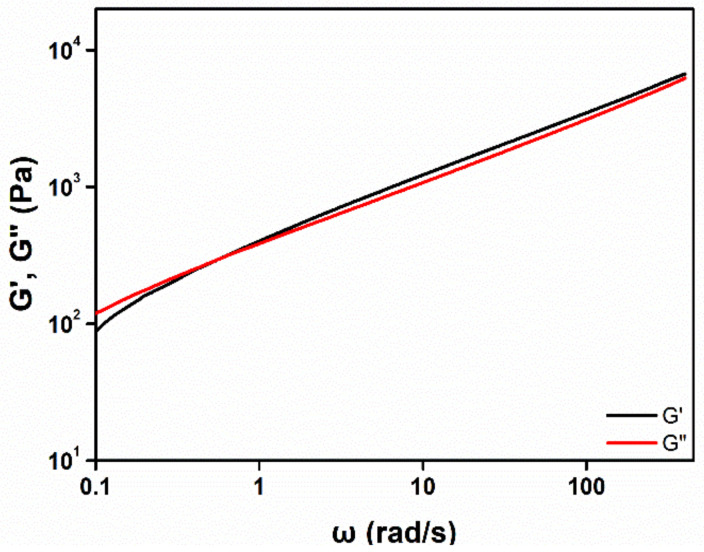
Storage modulus (G′) and loss modulus (G″) of the photo-reacted B-M-1.5-1.

Figure 5 shows the results of the viscoelasticity measurements of the photoreaction product of B-M-1.5-1 (viscoelasticity measurement results of all of the samples are shown in Figures S4 and S5). The storage modulus G′ and loss modulus G” represent the elasticity and viscosity, respectively. The elastic property dominated in the region where G′ was higher than G″, and the viscous property dominated in the region where G″ was higher than G′. In Figure 5, it can be seen that both G′ and G″ increased as the angular frequency increased. The sample had a G′/G″ crossover point around 0.6 rad/s, which indicated that the reaction product of B-M-1.5-1 had both viscosity and elasticity: in other words, viscoelasticity. The viscous nature of the product could improve the adhesion of the product to the end sides of the two glass slides or the coating matrix. In addition, the product could be sufficiently stretched due to its elastic property. B-M-1-2 and B-M-1-3 did not have a crossover point, and viscous properties were predominant in the whole angular frequency region, indicating that the two products were highly flowable liquids (Figure S4 and Table 1). Figure S6 shows the complex viscosity of the product samples. As the proportion of MMT-PDMS increased, the G′, G″, and complex viscosity tended to decrease (Figures S5 and S6). The photoreaction products with higher MMT-PDMS proportions showed lower elasticity and viscosity. It was observed that the B-M-1.5-1 with the widest MSW had an appropriate level of elasticity and viscosity.

### 3.2. Microencapsulation

**Figure 6:**
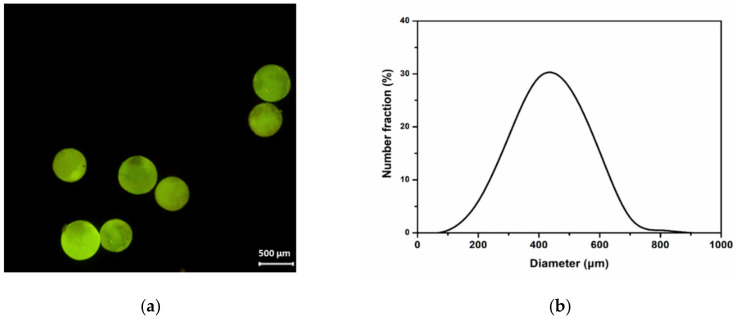
(**a**) A fluorescence micrograph of B-M-1.5-1-loaded microcapsules under 450–480 nm light. (**b**) Size distribution of the microcapsules.

**Figure 7:**
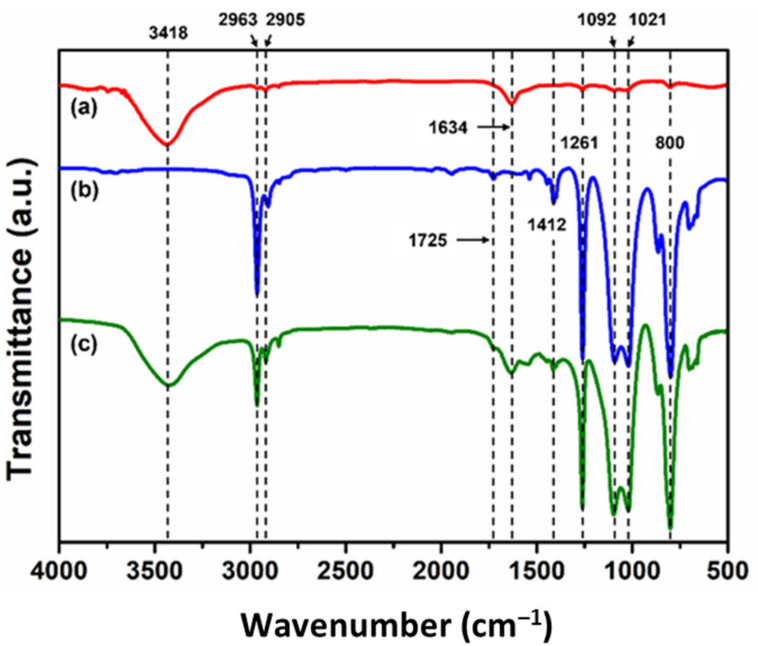
FT-IR spectra of (**a**) UF resin, (**b**) B-M-1.5-1, and (**c**) microcapsules.

B-M-1.5-1 was microencapsulated using an urea-formaldehyde (UF) resin as a shell material. Figure 6a displays the fluorescence micrograph of the microcapsules. For facile observation, 1 wt% of the fluorescent indicator (OIL-GLO 44-P) was added to the core material, and spherical microcapsules were observed under 450–480 nm light using a fluorescence microscope. The average capsule diameter was measured as 488 µm (Figure 6b). The formation of microcapsules was also confirmed by FT-IR spectroscopy. As shown in Figure 7a, the absorption bands of the UF resin were observed, including an -NH stretching vibration at 3418 cm^−1^ and a C=O stretching vibration at 1634 cm^−1^. In the spectrum of B-M-1.5-1 (Figure 7b), the following observations were made: C-H stretching vibration at 2963 and 2905 cm^−1^, C=O stretching vibration at 1725 cm^−1^, C=C stretching vibration at 1640 cm^−1^ (this band can be seen in an enlarged spectrum (Figure 2a)), CH_3_ bending vibration at 1412 cm^−1^, symmetrical deformation of the -CH_3_ in the -Si(CH_3_)_2_- group at 1261 cm^−1^, Si-O-Si stretching vibration at 1092 and 1021 cm^−1^, and Si-C stretching vibration at 800 cm^−1^. The characteristic absorption bands of UF resin and B-M-1.5-1 were all observed in the spectrum of the microcapsules (Figure 7c), confirming the formation of B-M-1.5-1-loaded microcapsules with UF resin as a shell material.

### 3.3. Primary Self-Healing Function

**Figure 8:**
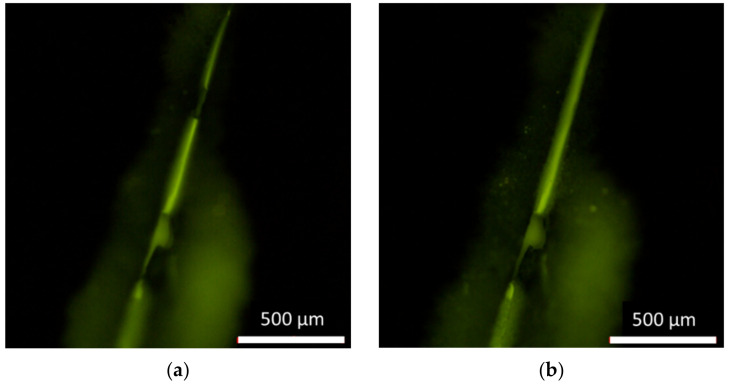
Fluorescence micrographs of the self-healing coating: (**a**) just after scratching and (**b**) after a few seconds.

For effective healing, when cracks occur in a microcapsule-type self-healing coating, the healing agent should be able to flow out of the microcapsules and fully fill the cracked region. To evaluate this function, a flowability test was conducted using the coating matrix containing B-M-1.5-1 microcapsules (Figure 8). OIL-GLO 44-P was added to B-M-1.5-1 as a fluorescence probe for facile observation. The microcapsules were dispersed in a commercial undercoating formulation at a concentration of 50 wt%. The resulting formulation was applied to a glass slide. After additional application of a top- coating formulation, the resulting self-healing coating was scratched using a razor blade with a 20-µm width. As soon as the scratch was generated, the core material was readily released. The damaged region was partly filled with the healing agent just after scratching (Figure 8a), and it was fully filled after a few seconds (Figure 8b).

**Figure 9:**
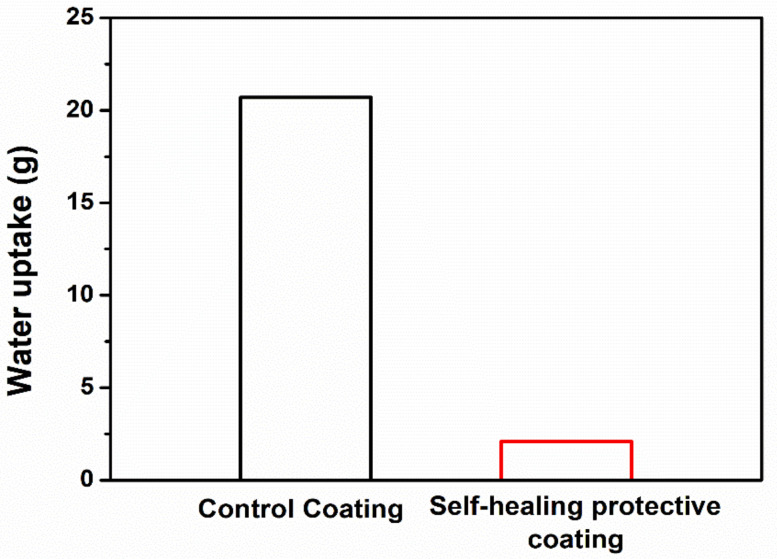
Water uptake graph of the coated mortars upon immersion of their cracked surface in water for 10 h.

**Figure 10:**
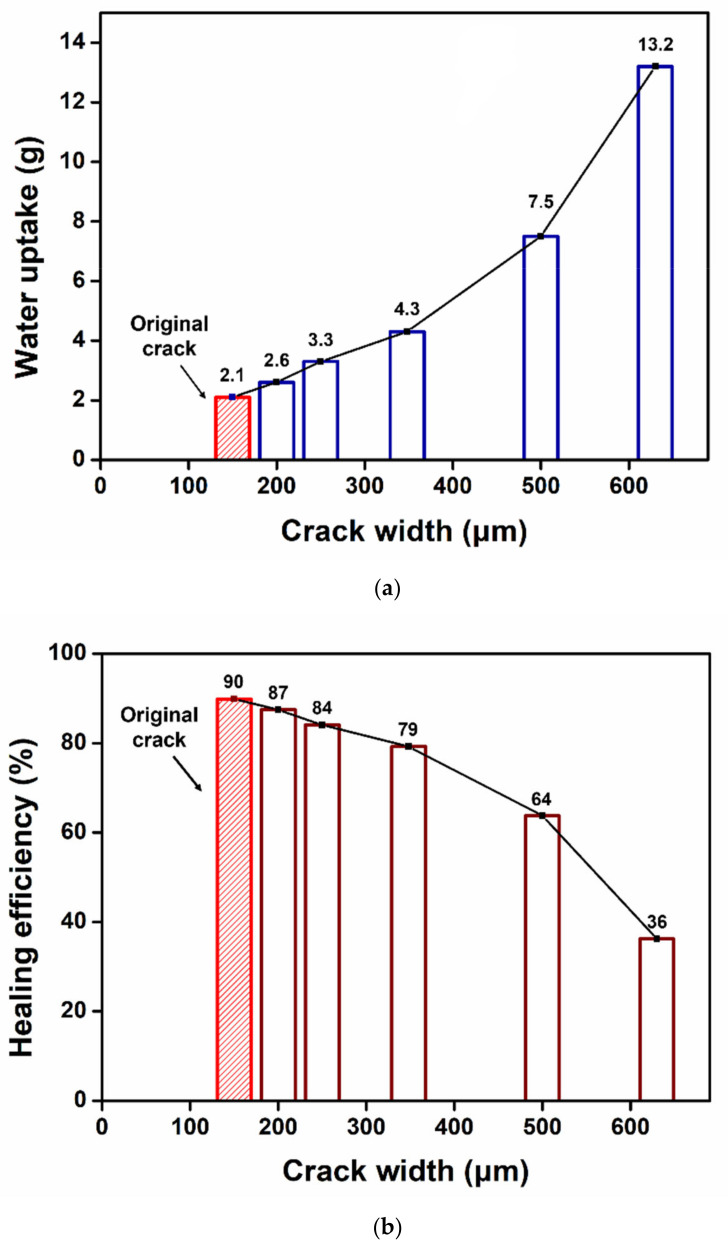
(**a**) Water uptake and (**b**) healing efficiency versus crack width of the self-healing protective coating containing B-M-1.5-1 microcapsules. The original cracks with a 150-µm mean width were expanded to 200, 250, 300, 500, or 630-µm width.

To evaluate the primary healing efficiency of the self-healing coating, a water sorptivity test was conducted. The B-M-1.5-1 microcapsules were dispersed in the commercial undercoating formulation. The resulting coating formulation was applied to the surface of the mortar specimens and then a top-coating was applied (Figure S2). A control coating without microcapsules was also prepared for comparison. Cracks with a 150-µm mean width were generated in both the control and self-healing coatings (Figure S7). The damaged region was photoirradiated to induce the photoreaction of the healing agent, and each coated side was immersed in water. The control coating specimen showed water uptake of 20.7 g, while the self-healing coating absorbed 2.1 g of the water (Figure 9). The healing efficiency was calculated to be 90% (Figure 10). The healing agent that was released from the ruptured microcapsules filled the cracked region and was converted into a semi-solid substance, which effectively blocked the water penetration. 

### 3.4. Capability of Maintaining the Healed State

The original crack with a 150-µm mean width was allowed to self-heal and was then expanded to a 200, 250, 300, 500, or 630-µm mean width using UTM by varying the speed and force (Figure 10). The water uptake increased with an increasing crack width (Figure 10a); therefore, the healing efficiency decreased as the crack width increased (Figure 10b). When the crack width was expanded to 250 µm, the healing efficiency decreased to 84%. However, the healing efficiency was maintained at around 80% even when the crack was expanded to a 350-µm width. This result indicated that the viscoelastic product of B-M-1.5-1 was stretched as the crack expanded, effectively maintaining the healed state. The healing efficiency further decreased to below 70% when the crack width was increased to 500 µm. This was due to the fact that the stretched film of the reacted healing agent was partially torn, losing its barrier function to some extent.

**Figure 11:**
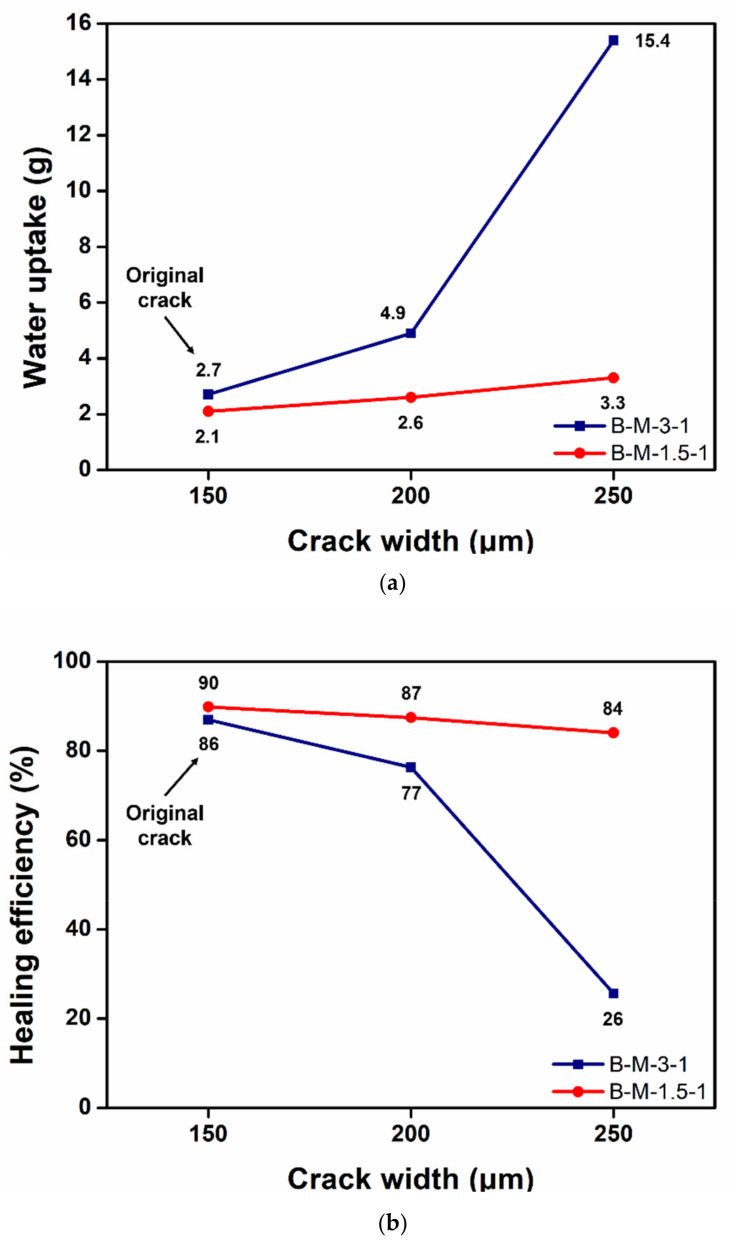
(**a**) Water uptake and (**b**) healing efficiency versus crack width of a self-healing protective coating. The mean width of the original crack was 150-µm. The original cracks were expanded to a 200 or 250-µm width.

For comparison, another water sorptivity test was conducted using B-M-3-1 that showed a much lower MSW than B-M-1.5-1 (Figure 4). B-M-3-1 was microencapsulated, and the self-healing coating containing the microcapsules was coated onto the mortar specimens. The original crack with a 150-µm mean width was allowed to self-heal, and it was then expanded to a 200 or 250-µm width. As the crack width was increased, the water uptake drastically increased (Figure 11a), indicating a significant reduction in the healing efficiency (Figure 11b). While more than 80% of the healing efficiency was maintained in the case of the B-M-1.5-1 coating at a 250-µm crack width, the B-M-3-1 coating could not maintain its healed state upon crack expansion. This result was attributed to the lower stretchability of B-M-3-1 than B-M-1.5-1 (Figure 4). Because the reaction product of B-M-3-1 shows a smaller MSW than that of B-M-1.5-1, crack expansion to a 200-µm width would result in a larger degree of tearing of the B-M-3-1 product film, compared to B-M-1.5-1 product film. The tearing of the B-M-3-1 product film would have proceeded drastically when the crack was further expanded to a 250-µm width. Much more water penetrated through the enlarged hole of the B-M-3-1 product film, resulting in significant water uptake increase.

## 4. Conclusions

The combined mixtures of BMT-PDMS, MMT-PDMS, and BIE provided viscoelastic substances by UV irradiation. Their viscoelasticity could be controlled by varying the mass ratio of BMT-PDMS and MMT-PDMS. By conducting the stretchability test of the reacted mixtures, a 1.5:1 mass ratio of BMT-PDMS and MMT-PDMS was determined to be the optimal composition because the mixture showed the widest MSW. Microencapsulation of the selected healing agent mixture (B-M-1.5-1) with the optimal composition was conducted by an in situ polymerization method. Microcapsules loaded with the healing agent were prepared and dispersed in an undercoating formulation to obtain a self-healing coating on mortar specimens. Initial cracks with a width of around 150-μm were created in the coatings, and the cracks were then expanded up to a width of 650 μm. By conducting a water sorptivity test at each crack width, the self-healing efficiency and capability of maintaining the healed state of the coating system were evaluated. The B-M-1.5-1-based coating showed a healing efficiency of 90% at a 150-μm crack width and maintained a healing efficiency of about 80% up to a crack width of 350-μm. This self-healing coating is a promising system for the protection of structural materials that can undergo crack formation and expansion.

## Figures and Tables

**Table 1:** Mass ratio and the state of the photoreaction product of BMT-PDMS and MMT-PDMS Mixtures.

Sample Code ^a^	BMT-PDMS	MMT-PDMS	State of Photoreaction Product
B-M-3-1	3	1	Semi-solid
B-M-2-1	2	1	Semi-solid
B-M-1.5-1	1.5	1	Semi-solid
B-M-1-1	1	1	Semi-solid
B-M-1-1.5	1	1.5	High-viscous liquid
B-M-1-2	1	2	Low-viscous liquid
B-M-1-3	1	3	Low-viscous liquid

^a^ Each sample contained 1 wt% of BIE and 1 wt% of a fluorescent fluid.

## Data Availability

Not applicable.

## Supplementary Materials

The following are available online at https://www.mdpi.com/article/10.3390/ma14206198/s1: Scheme S1: Chemical structure and photocrosslinking of BMT-PDMS and MMT-PDMS; Figure S1: Schematic illustration of the stretchability test; Figure S2: Schematic illustrations of water sorptivity test; Figure S3: FT-IR spectra of B-M-1-1 (a) before and (b) after photoirradiation; Figure S4: Storage modulus (G′) and loss modulus (G″) of the following reaction products: (a) B-M-3-1, (b) B-M-2-1, (c) B-M-1.5-1, (d) B-M-1-1, (e) B-M-1-1.5, (f) B-M-1-2, and (g) B-M-1-3; Figure S5: (a) Storage modulus (G′) and (b) loss modulus (G″) of the reaction products; Figure S6: Complex viscosity (η*) curves of the reaction products; Figure S7: A microscopic image of the crack generated in the coated mortar specimens; and Table S1: Maximum stretched widths (MSWs) of the reaction products according to the mass ratio of the two methacrylates.
