# Supplementary material for: Microcapsule-Type Self-Healing Protective Coating That Can Maintain Its Healed State upon Crack Expansion
Source: Materials (Basel). 2021 Oct 19;14(20):6198. doi: 10.3390/ma14206198 (PMC8539965; doi:10.3390/ma14206198)
Supplement: Supplementary file 1 [file materials-14-06198-s001.zip › materials-1404823-supplementary.pdf]

<Supplementary Materials>

Microcapsule-Type Self-Healing Protective Coating That Can Maintain the Healed State upon Crack Expansion

Ji-Sun Lee, Hyun-Woo Kim, Jun-Seo Lee, Hyun-Soo An and Chan-Moon Chung *

Department of Chemistry, Yonsei University, Wonju, Gangwon-do 26493, Korea

*Correspondence: cmchung@yonsei.ac.kr; Tel.: +82-33-760-2266

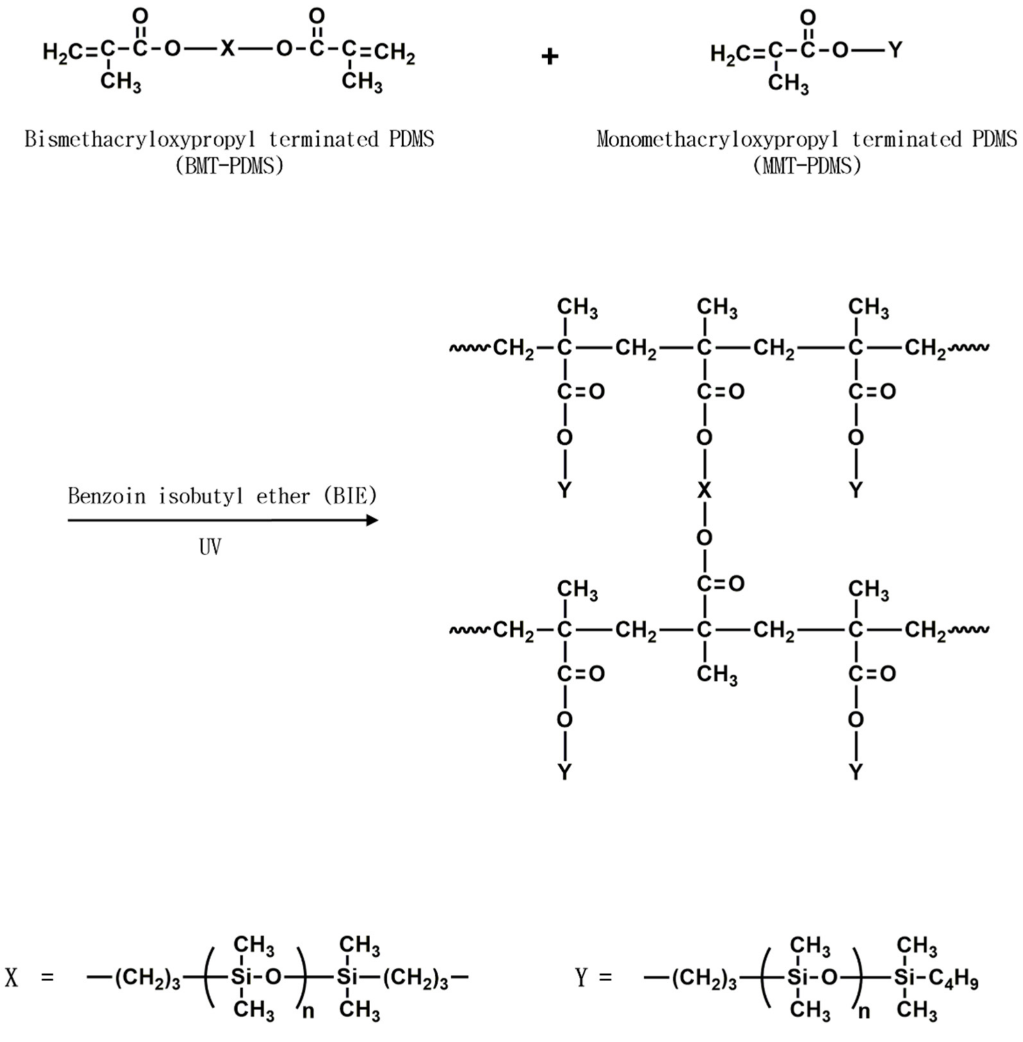

Scheme S1. Chemical structure and photocrosslinking of BMT-PDMS and MMT-PDMS.

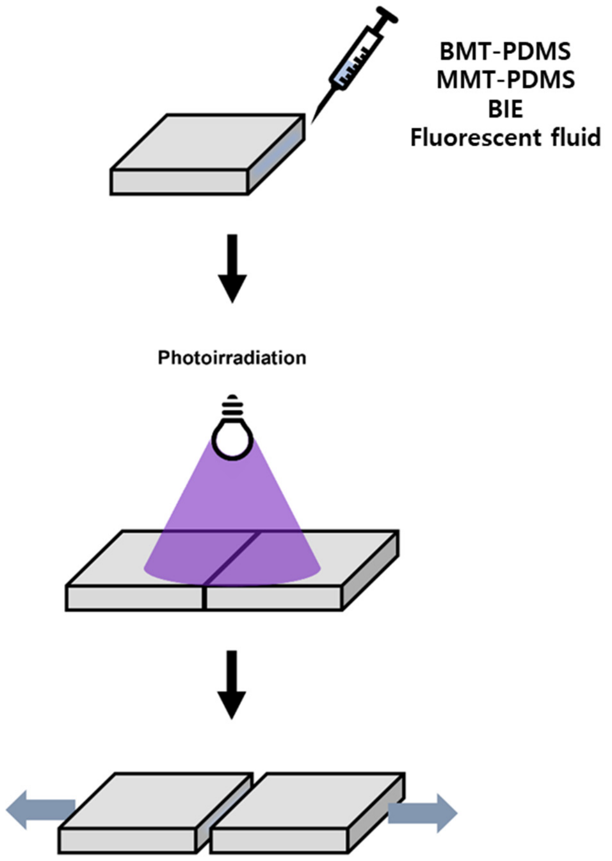

Figure S1. Schematic illustration of the stretchability test.

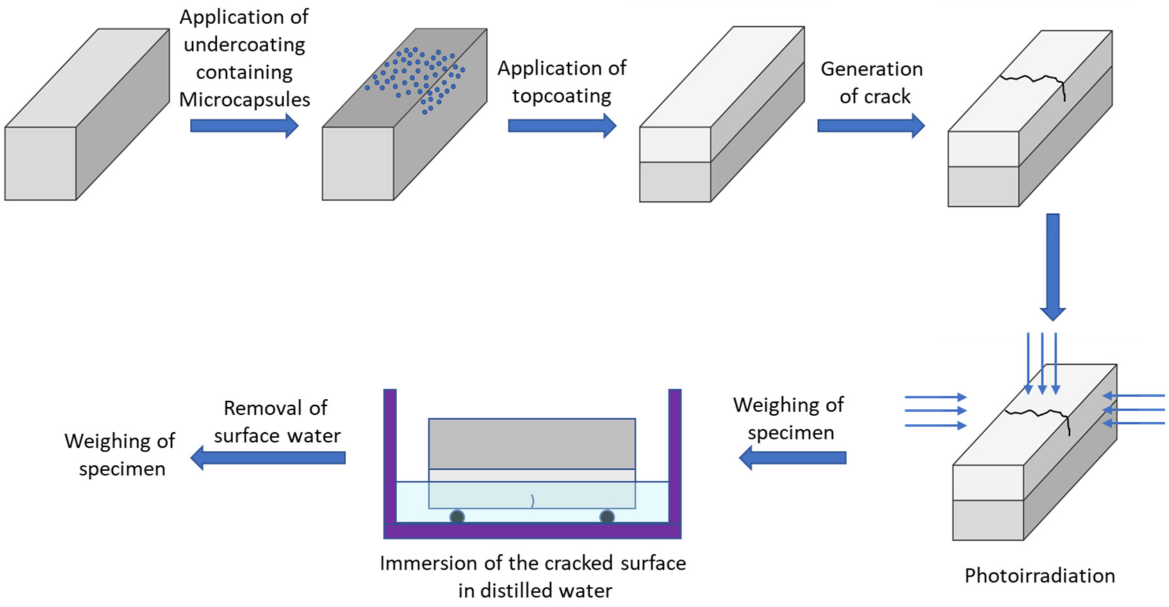

Figure S2. Schematic illustration of water sorptivity test.

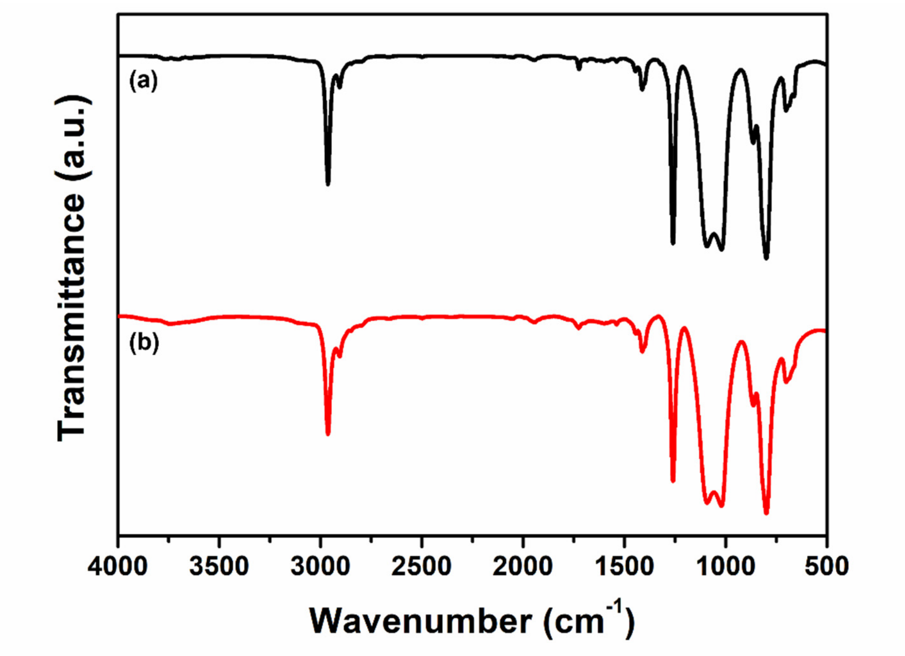

Figure S3. FT-IR spectra of B-M-1-1 (a) before and (b) after photoirradiation.

Table S1. Maximum stretched widths (MSWs) of the reaction products according to mass ratio of two methacrylates [a].

	(a)	(b)	(c)	(d)	(e)	(f)	(g)
MSW (mm)	1.20	1.22	1.62	1.10	0.79	-	-

[a] Mass ratio of BMT-PDMS and MMT-PDMS: (a) 3:1; (b) 2:1; (c) 1.5:1; (d) 1:1; (e) 1:1.5; (f): 1:2; (g) 1:3.

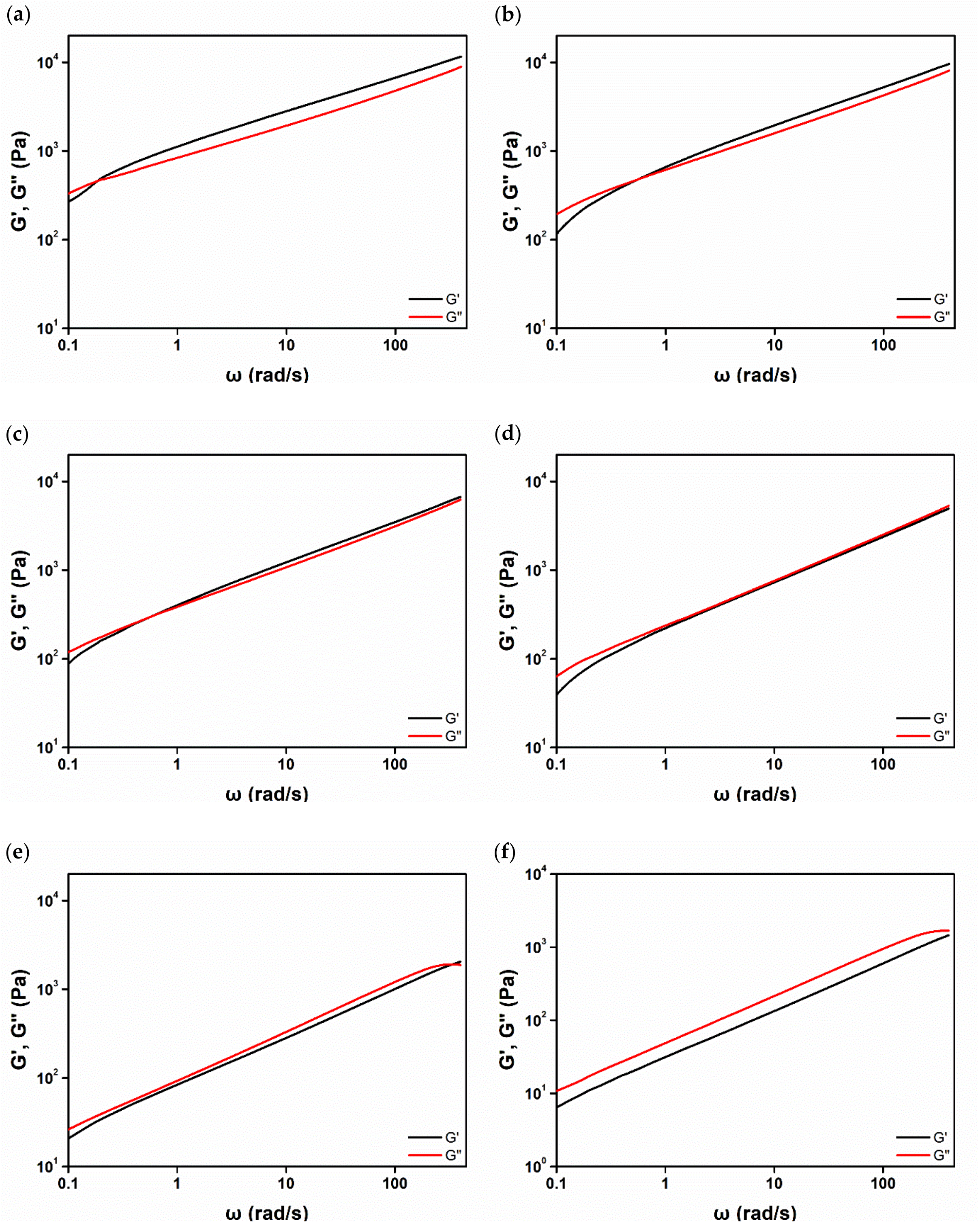
(a)
(b)
(c)
(d)
(e)
(f)
G', G'' (Pa)
ω (rad/s)
G'
G''
0.1
1
10
100

(g)

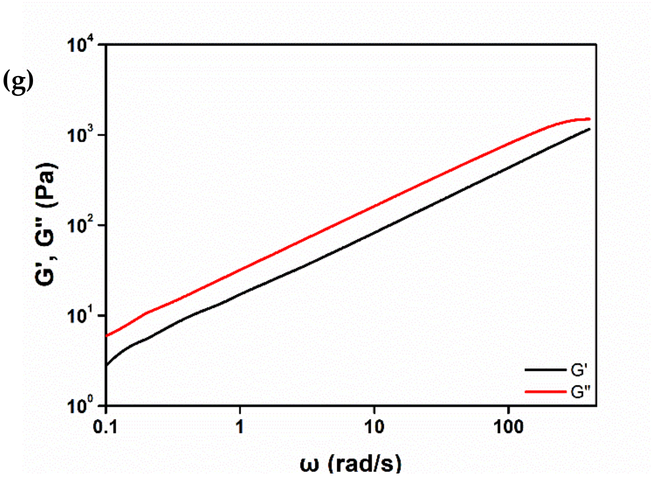

Figure S4. Storage modulus (G′) and loss modulus (G″) of the reaction products: (a) B-M-3-1; (b) B-M-2-1; (c) B-M-1.5-1; (d) B-M-1-1; (e) B-M-1-1.5; (f) B-M-1-2; (g) B-M-1-3.

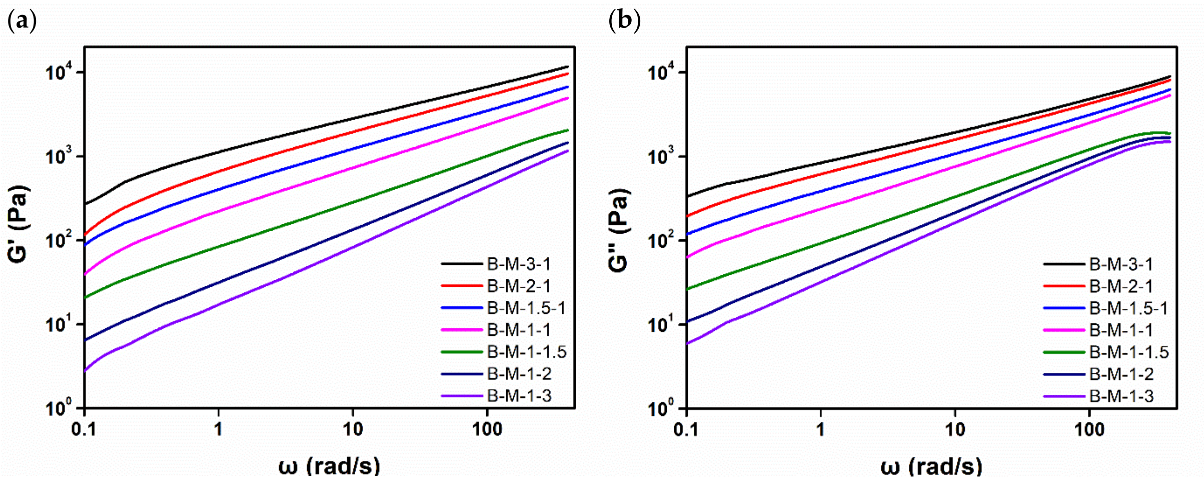

Figure S5. (a) Storage modulus (G′) and (b) loss modulus (G″) of the reaction products.

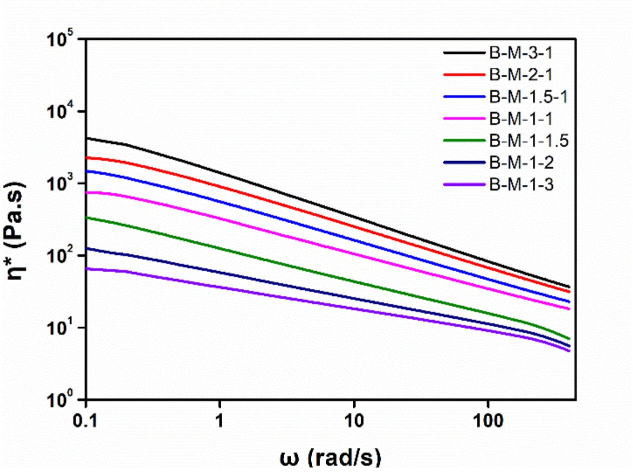

Figure S6. Complex viscosity (η*) curves of the reaction products.

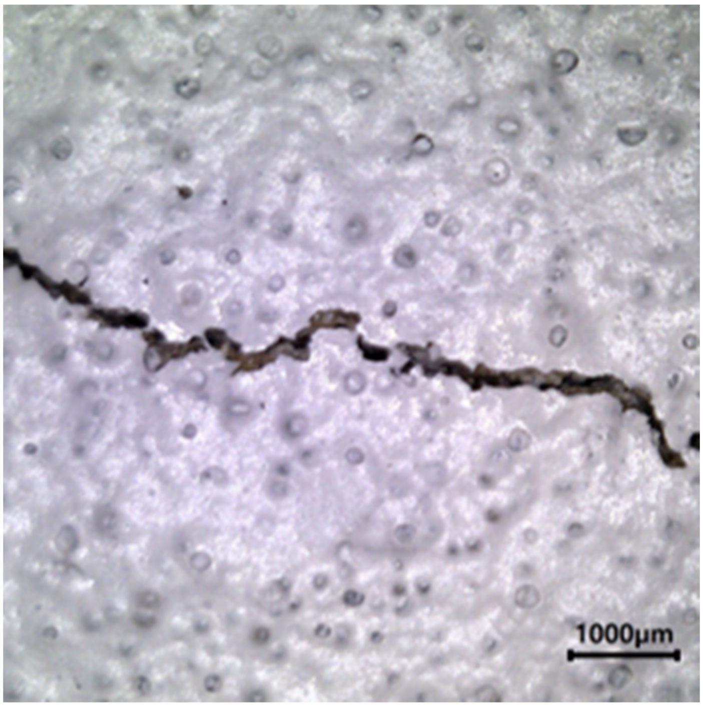

Figure S7. A microscopic image of a crack generated in the coated mortar specimens.
